# Smart Inhalation Therapy: Boosting siRNA Efficacy with Inulin-Based Multifunctional Polymers

**DOI:** 10.1021/acsami.5c18977

**Published:** 2025-10-31

**Authors:** Salvatore E. Drago, Marta Cabibbo, Cinzia Scialabba, Emanuela F. Craparo, Gennara Cavallaro

**Affiliations:** Lab of Biocompatible Polymers, Department of Biological, Chemical and Pharmaceutical Sciences and Technologies (STEBICEF), 18998 University of Palermo, Via Archirafi 32, Palermo 90123, Italy

**Keywords:** inulin, polymethyloxazoline, siRNA, lung delivery, muco-diffusion

## Abstract

Therapeutic delivery of siRNA via inhalation holds significant promise for managing severe pulmonary diseases. However, an effective delivery platform capable of overcoming the lung’s physical and biological barriers is essential to achieve efficient gene silencing in the airway epithelium. Here, we describe the synthesis of an inulin (INU)-based copolymer, INU-VS-*g*-(PMeOx; bAPAE), designed for siRNA inhalation. A stepwise synthesis was employed: first, INU was functionalized with divinyl sulfone to form INU-VS, allowing controlled conjugation of 1,2-bis­(3-aminopropylamino)­ethane (bAPAE) and poly­(2-methyl-2-oxazoline) (PMeOx) at, respectively, 25 and 5 mol % on the total INU repeat units. The resulting copolymer exhibited protonatable amine groups essential for nucleic acid complexation. Stable formation of siRNA polyplexes was found at low polymer/siRNA weight ratios (*R* = 5), with a mean size below 30 nm. Potentiometric titrations confirmed efficient buffering capacity while fluorescence microscopy demonstrated pH-dependent membrane destabilization, indicating an enhanced endosomal escape potential at low pH values. Stability studies in mucus and pulmonary surfactant revealed that polyplexes remained intact even at high mucin concentrations (5 mg mL^–1^) and exhibited high muco-diffusivity. Biocompatibility assessments on 16-HBE showed excellent cytocompatibility with over 80% cell viability even at high polymer concentrations. Uptake studies confirmed polyplex internalization and siRNA release. Experiments on MDA-MB-231-eGFP cells demonstrated siRNA-mediated silencing. Overall, together with the excellent aerosol performance of the polyplex aqueous dispersions, these findings highlight the potential of INU-VS-*g*-(PMeOx; bAPAE) as a versatile and effective siRNA carrier for pulmonary administration, paving the way for future therapeutic applications in respiratory diseases.

## Graphical abstract

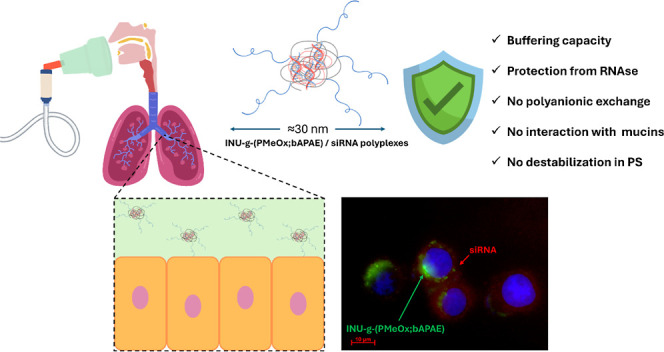

## 1 Introduction

Cystic fibrosis (CF), chronic obstructive pulmonary disease (COPD), and asthma are lung diseases characterized by chronic inflammation and progressive lung dysfunction, which severely affect patients’ quality of life^1^ causing around 4 million deaths per year.^2^ Effective treatments for these diseases are still lacking;^3,4^ current therapies are focused to manage symptoms and slow down progression of such pathologies.

Studies focusing on such diseases have highlighted the overexpression of specific genes,^5^ which has sparked growing interest in the use of siRNA-based therapies, especially after the approval of the first siRNA-based therapy, Onpattro, in 2018. Treatment of lung diseases by inhaled siRNA-based therapies could enable high local concentrations of RNA in the lungs while reducing systemic degradation^3^ and minimizing side effects.^6^ Preclinical studies on aerosolized RNA have shown encouraging results.^7–10^

However, inhaled RNA-based formulations require specific features to reach deep regions of the respiratory tract, such as aerodynamic diameter between 0.5 and 5 μm, and to overcome pulmonary obstacles, such as natural barriers and clearance mechanisms, which often hinder efficient delivery.^11,12^ The first barrier encountered is the mucus layer lining the airways,^13^ a hydrogel that traps inhaled particles through steric hindrance and adhesive interactions (electrostatic, hydrophobic, and hydrogen bonding),^14^ aiding their removal via mucociliary clearance (MCC).^11^ In the alveolar region, lung surfactant components are another barrier to siRNA-based formulations^15,16^ because they can adsorb onto particles, altering their in vivo fate.^17^ Furthermore, the bronchial epithelium, that often is the cell target of anti-inflammatory therapies,^18,19^ is a barrier itself due to tight junctions and low endocytic activity.^20^

All these challenges are extremely variable depending on the disease severity and type, where increased mucus viscosity and mucin hypersecretion typical in COPD and CF further hinder delivery.^21–23^ Disease progression also reduces pulmonary inspiration function, limiting the efficiency of inhalation-based therapies.

Therefore, for efficient inhalation delivery of siRNA, nanosized delivery systems are suitable carriers to allow penetration through the mucus layer and protection of siRNA from nuclease degradation, enhancing cellular uptake^24^ and extending its residence time in the lungs.^25,26^ Among them, polymer-based nanocarriers offer biocompatibility, stability, and versatility.^27,28^ These polymer materials are typically synthesized to exhibit positive charges, which enable them to bind to nucleic acids via electrostatic interactions.^29^

A key strategy to minimize interactions of polyplexes with lung fluids (mucus and surfactant) is to modulate the carrier hydrophilicity by grafting hydrophilic polymers as the gold standard poly­(ethylene glycol) (PEG).^30,31^ Among other similar materials, poly­(2-oxazoline)­s (POx), a class of nontoxic and biocompatible polymers with pseudopolypeptide structures, have shown to confer stealth-like properties like PEG,^32–34^ with the added benefit of fast clearance and lower tissue accumulation.^35,36^

Considering these factors, this study focuses on the development of an inhalable formulation for siRNA, based on polyplexes between a polymeric carrier and siRNA. The polymeric component was synthesized starting from inulin (INU), a natural, water-soluble, biocompatible, nonimmunogenic, and nonantigenic polysaccharide, already used to develop high-performance polymeric gene vectors.^37,38^ To impart cationic charge and to enhance hydrophilicity, 1,2-bis­(3-aminopropylamino)­ethane (bAPAE) and poly­(2-methyloxazoline) (PMeOx) were grafted, respectively, to INU. The potential of the obtained new semisynthetic graft copolymer as a siRNA carrier for inhalation therapy was evaluated in terms of interactions of obtained polyplexes with lung fluid components, ability to be internalized into bronchial epithelial cells, as well as ability to silence gene expression.

## 2 Results and Discussion

### 2.1 Synthesis and Characterization of the INU-VS-*g*-(PMeOx; bAPAE) Copolymer

Synthetic polymers represent valid candidates as siRNA carriers, since structural and functional properties can be tailored to provide specific features required for the complexation of a specific siRNA sequence.^29^

Here, the synthesis of a novel inulin (INU)-based copolymer as a siRNA carrier for pulmonary administration by inhalation was described. The choice of INU as starting polymer was made being a natural polysaccharide widely used for application in nanomedicine and regenerative medicine.^39^ To confer the ability of complex genetic material through electrostatic interactions, we selected tetramine 1,2-bis­(3-aminopropylamino)­ethane (bAPAE) as the functional moiety. As hydrophilic moieties, aimed at reducing interactions with lung mucus components to the resulting polycation,^40,41^ we chose poly­(2-methyl-2-oxazoline) (PMeOx), which was synthesized via Cationic Ring Opening Polymerization (CROP) in experimental conditions to obtain a resulting polymer with a ${\mathrm{M̅}}_{w}$ of 5 kg/mol (Figure S1).

To allow an easy and suitable covalent grafting of proper amount of either bAPAE or PMeOx, INU was, in a first step, modified in DMF by covalent derivatization with divinyl sulfone (DVS) (Scheme 1, step a) to slow down reaction kinetics and prevent cross-linking of the polymer chains.

**Scheme 1:**
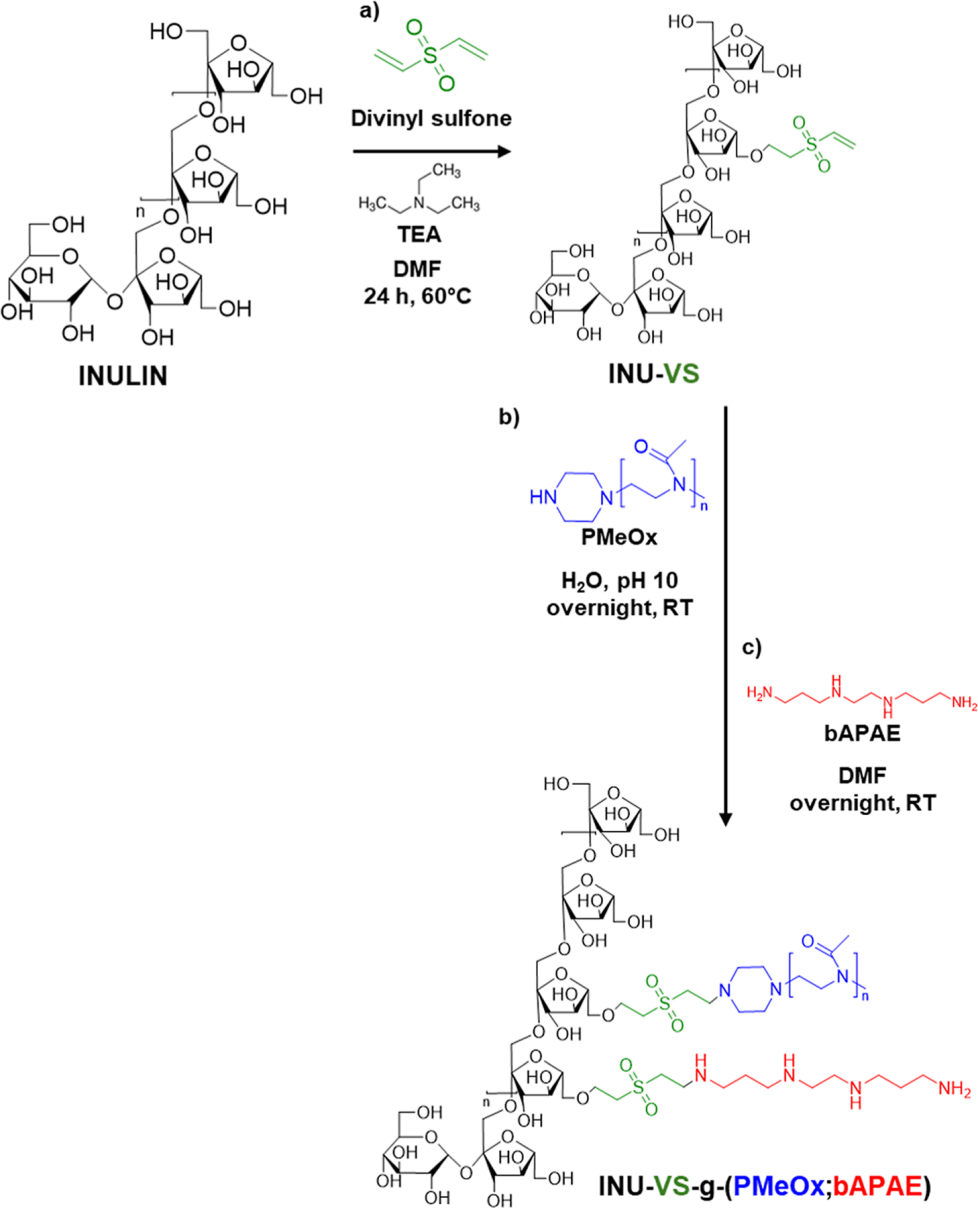
Synthetic Procedure to Obtain INU-VS (Step a) and INU-VS-*g*-(PMeOx; bAPAE) (Step b and c)

The degree of derivatization of the resulting INU-vinyl sulfone (INU-VS) derivative was 29.8 ± 1.4 mol %, as determined by ^1^H NMR spectroscopy (Figure 1, spectrum a) by comparing the peaks of the vinyl groups (6.8, 6.4, and 6.3 ppm) with those of the INU repeat units (4.3–3.5 ppm).

**Figure 1:**
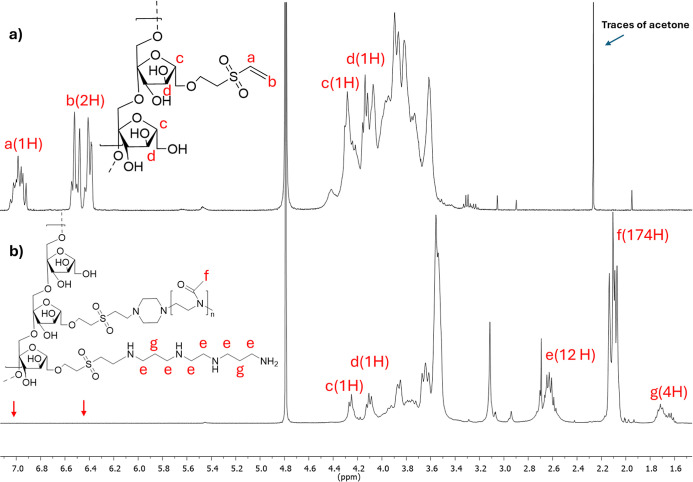
^1^H NMR spectra of (a) INU-VS and (b) INU-VS-*g*-(PMeOx; bAPAE) copolymers in D_2_O.

The final INU-VS-*g*-(PMeOx; bAPAE) copolymer was obtained through an easy telescoping synthesis, by first mixing the INU-VS derivative with PMeOx in an aqueous medium (Scheme 1, step b) and then transferring the reaction mixture to an organic solution (DMF) containing an excess of bAPAE (Scheme 1, step c), needed to prevent cross-linking, as the oligoamine used contains two primary amino groups (–NH_2_).

This strategy to exploit the vinyl sulfone residues for the conjugation of tetramine to the polymer backbone allows preservation of the amino functionality within the bond thanks to the conversion of the primary amine of the bAPAE into a secondary amine. In fact, the loss of the amine group is obtained by following other synthetic approaches which involve the use of bis­(4-nitrophenyl) carbonate or similar, due to amide formation.^30^ This is a great advantage as it allows for an extra amine group in each functionalized repeat unit, implementing siRNA complexation and buffering properties. Under the used experimental conditions, a degree of derivatization in PMeOx (DD_%PMeOx_) and in bAPAE (DD_%bAPAE_) of about 4.7 ± 1.2 mol % and 24.8 ± 1.2 mol %, respectively, were obtained and reported in Table 1. These values were calculated by ^1^H NMR analysis in D_2_O (Figure 1, spectrum b) by relating the integral of the signals that are attributable to 4 protons of bAPAE (at δ 1.72 ppm) or the signals corresponding to 174 protons of PMeOx (at δ 2.1 ppm) with the integral of the signal corresponding to the 2 protons of the INU repeat unit (at δ 4.2 and 4.1 ppm).

**Table 1:** Weight-Average Molecular Weight ( ${\mathrm{M̅}}_{w}$ ), Weight-Average Molecular Numeric Weight ( ${\mathrm{M̅}}_{n}$ ), Polydispersity Index ( ${\mathrm{M̅}}_{w}/{\mathrm{M̅}}_{n}$ ), and Chemical Composition in Terms of DD in VS, PMeOx, and bAPAE of Obtained Copolymers

	molecular weight			derivatization degree (DD %)		
copolymers	${\mathrm{M̅}}_{w}$ (g/mol)	${\mathrm{M̅}}_{n}$ (g/mol)	$${\mathrm{M̅}}_{w}/{\mathrm{M̅}}_{n}$$	DD_VS_	DD_PMeOx_	DD_bAPAE_
INU	3900	3300	1.16			
INU-VS	5300	4100	1.30	29.8 ± 1.4		
INU-VS-*g*-(PMeOx; bAPAE)	25,400	15,300	1.65		4.7 ± 1.2	24.8 ± 1.2

We observe in the spectrum b that the peaks of the vinyl groups (6.98, 6.52, and 6.41 ppm) are no longer noticeable, indicating that all VS residues in the INU-VS have been fully functionalized with PMeOx and bAPAE. Moreover, the SEC analysis (Table 1) showed a ${\mathrm{M̅}}_{w}$ of the INU-VS-*g*-(PMeOx; bAPAE) copolymer approximately of 15 kg/mol, which is consistent with the theoretical one calculated based on the VS, PMeOx, and bAPAE functionalization degree.

Therefore, the chosen synthetic procedure is very versatile, exploiting the previous functionalization with VS to obtain a new hydrophilic and cationic copolymer in one pot, which offers a very high-performance reaction in terms of grafting of amino groups and PMeOx chains.

In particular, with a simple and easy synthetic procedure, a new cationic and hydrophilic INU derivative was obtained, in which about 25 mol % of the repeat units have four amino groups, three secondary and one primary, protonatable as a function of pH. These functions are essential for both nucleic acid complexation and imparting buffering properties, which play a crucial role in facilitating endosomal escape via the proton sponge effect.^42^

To study the buffering properties of INU-VS-*g*-(PMeOx; bAPAE), potentiometric titrations were carried out (Figure S2). The titration curve of INU-*g*-(PMeOx; bAPAE) exhibits the characteristic profile of a polyprotic base, showing a gradual reduction in the slope between pH 7.5 and 5. This indicates that the polymer provides moderate, distributed buffering with the strongest activity occurring in the pH range where secondary amine protonation predominates. The absence of a sharp inflection point suggests that the polymer lacks a single dominant p*K*_a_, instead featuring multiple weakly basic sites that contribute to a broad buffering region.

In the pH range of 7 to 5, critical for endosomal escape via the proton sponge effect,^43^ INU-VS-*g*-(PMeOx; bAPAE) exhibited a buffering capacity (β), expressed as μmol HCl/ΔpH, of 16.5 (i.e., 0.55 per mg), which is higher than that measured for bAPAE under the same conditions (β_bAPAE_ = 7.5). This difference could be attributed to the transformation of the primary amines into a secondary amine due to the involvement in bonding with VS residues, with a p*K*_a_ different from the original one.

According to the literature,^44^ within the same pH range, PEI and PAMAM exhibit β values of approximately 4.8 and 5.7 per mg of material, respectively. In contrast, the polymer presented in this work shows a β value of 0.55 per mg of material, which is about ten times lower. However, it should be noted that this polymer is multifunctional, and the amine-containing fraction represents only about 10% of the total mass, while the remaining components (inulin and PMeOx) are nonionic species and do not contribute to buffering.

To better investigate the protonation state as a function of pH, the backward titration was carried out (Figure 2a), and the data were elaborated according to the De Levie method,^45^ considering the activity corrections through the Davies expression [Disp-formula eq1]

1 $$\log y=-0.5\left(\frac{\sqrt{I}}{1+\sqrt{I}}-0.3I\right)$$

where *y* is the activity coefficient and *I* is the ionic strength.

**Figure 2:**
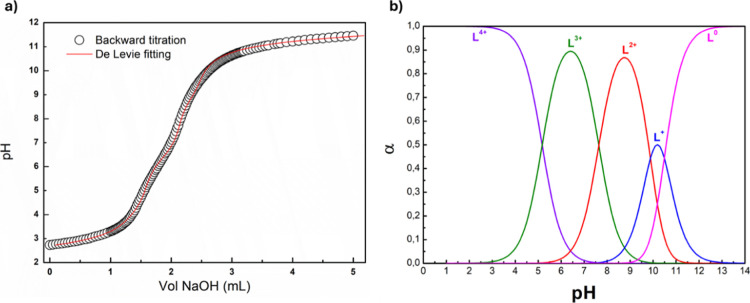
(a) Backward acid–base titration (NaOH volume versus pH) of INU-VS-*g*-(PMeOx; bAPAE) and De Levie fitting curve and (b) speciation curves (α versus pH) for each α of INU-VS-*g*-(PMeOx; bAPAE).

For this titration, the fitting function obtained is the following [Disp-formula eq2]

2 $$V_{B}=-\frac{V_{0}\left[C_{0}\left(4\alpha_{4}+3\alpha_{3}+{2\alpha }_{2}+\alpha_{1}\right)+\Delta \right]+V_{A}\left(\Delta -C_{A}\right)}{\Delta +C_{B}}$$

3 $$\Delta =C_{H^{+}}-\frac{K_{W}}{C_{H^{+}}\cdot y^{2}}+C_{B}$$

where *V*_A_ and *C*_A_ are the volume and molarity of HCl used for the forward titration, respectively, while *V*_B_ and *C*_B_ are the volume and molarity of NaOH for the backward titration, respectively, *V*_0_ is the volume of INU-VS-*g*-(PMeOx; bAPAE) sample, *C*_0_ is the equivalent bAPAE molarity while $\alpha_{4}{,\alpha_{3},\alpha }_{2},\alpha_{1}$ are the protonation degree, *K*_W_ is the dissociation constant of water, and $C_{H^{+}}$ is the H^+^ concentration.

Through curve fitting analysis, the p*K*_a_ values of the amine groups in the polymer were found to be equal to 10.49, 9.88, 7.63, and 5.17, and from these, the speciation curves were subsequently determined. As shown in Figure 2b, at pH 7.4, the triprotonated species (L^3+^) and diprotonated species of the copolymer accounted for approximately 63% and 36%, respectively. In acidic conditions, the proportion of the tetraprotonated species (L^4+^) gradually increases, becoming the dominant species at around pH 5 (approximately 60%). This behavior may influence the copolymer’s interaction with biological membranes, depending on the intracellular compartment, specifically cytosolic (pH 7.4) and endosomal (pH 5). This pH-dependent interaction suggests a potential mechanism that facilitates endosomal escape.

To investigate this, an in vitro study was performed using human bronchial epithelial cells (16-HBE) as a model of biological membranes. An aqueous solution of the INU-VS-*g*-(PMeOx; bAPAE) graft copolymer was incubated at two different pH values: 7.4 and 5. Yellow Oxazole was used as a fluorescent probe to differentiate healthy cells from those undergoing apoptosis or already dead based on increased membrane permeability.

As shown in Figure 3a higher internalization of the fluorescent probe, indicative of membrane destabilization, was observed only at pH 5. This confirms that the copolymer can act as a membrane-permeabilizing agent specifically under acidic conditions, likely due to changes in protonation states.^46,47^ This property provides an additional mechanism that promotes the endosomal escape process.

**Figure 3:**
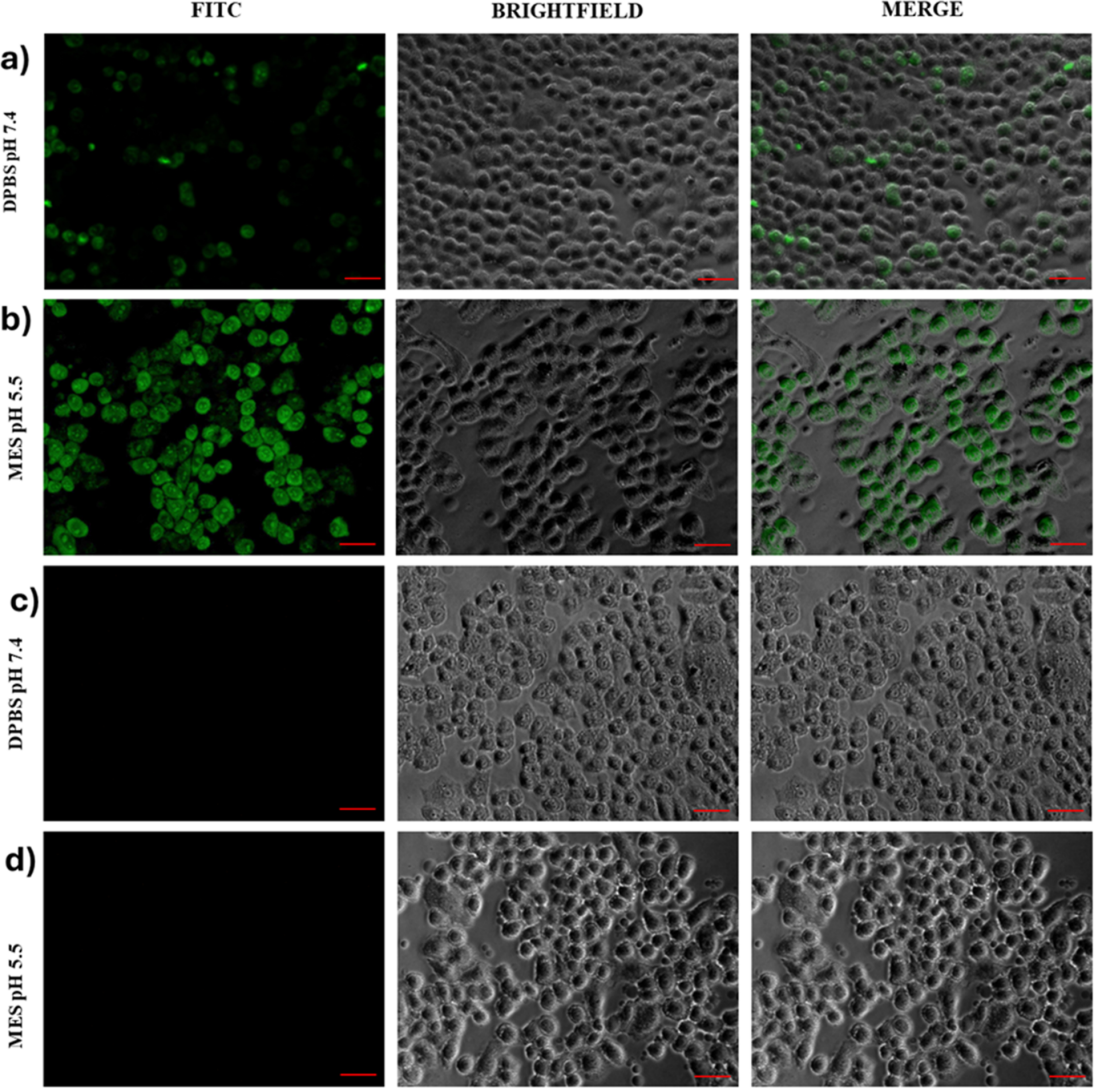
Fluorescence images of 16-HBE cells after incubation for 20 min in DPBS at pH 7.4 (a,c) and in MES at pH 5.5 (b,d), with or without the INU-VS-*g*-(PMeOx; bAPAE) graft copolymer, respectively.

### 2.2 siRNA–Copolymer Complexation Studies and Characterization of Obtained Polyplexes

To evaluate the ability of the synthesized copolymer to complex siRNA, equal volumes of aqueous dispersions containing siRNA at 0.2 mg mL^–1^ and INU-VS-*g*-(PMeOx; bAPAE) copolymer at increasing concentrations were mixed to obtain different polymer/siRNA weight ratios (*R*); agarose gel electrophoresis (Figure 4a) and dynamic light scattering (DLS) measurements (Figure 4b) were conducted on the resulting polyplexes.

**Figure 4:**
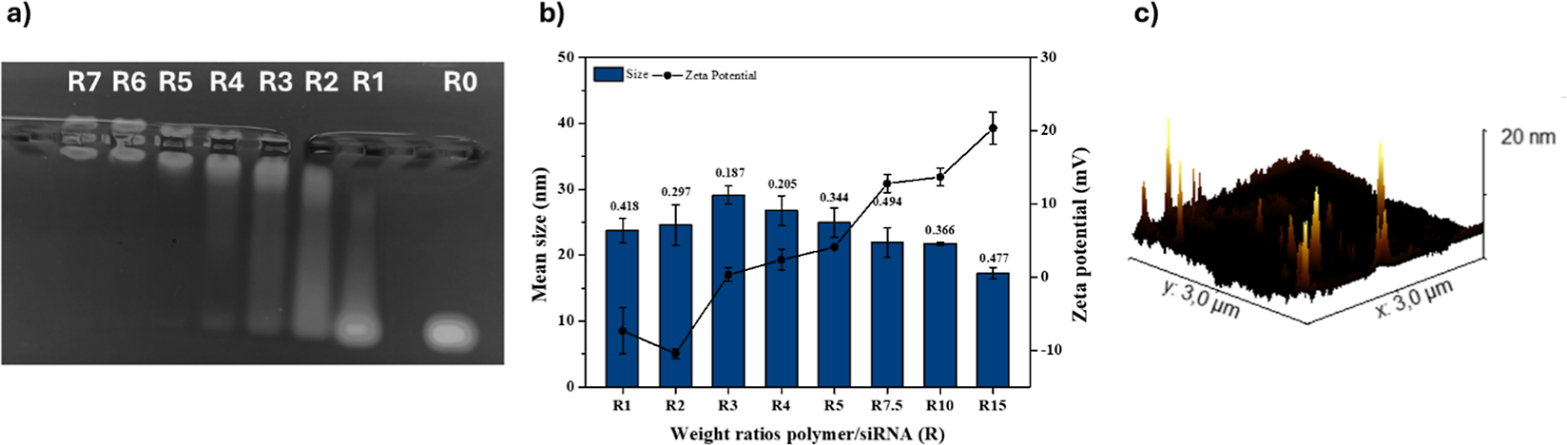
(a) Agarose gel electrophoresis of INU-VS-*g*-(PMeOx; bAPAE)/siRNA polyplexes obtained in PBS at various copolymer to siRNA weight ratios (*R*) ranging between 0, 1, 2, 3, 4, 5, 6, and 7; (b) mean size (histogram), PDI values (data labels), and zeta potential (continued line) of INU-VS-*g*-(PMeOx; bAPAE)/siRNA polyplexes at *R* ranging between 0 and 15, in HEPES buffer at pH 7.4 (data are reported as means ± SD, *n* = 3); and (c) AFM image of polyplexes obtained at *R* 15.

As it can be seen in Figure 4a, the INU-VS-*g*-(PMeOx; bAPAE) copolymer was able to stably complex siRNA starting from *R* = 5; considering the protonated polymer species at pH 7.4 (Figure 2b, this value corresponds to an N/P ratio of about 2.1. As expected (Figure 4b), the zeta potential increases as *R* increases, starting from negative values at *R* = 1 and reaching slightly positive values (approximately +20 mV) at *R* = 15; while mean size was below 30 nm at all *R* values, demonstrating that no aggregation occurs, even when the zeta potential is near neutrality. This behavior can be attributed to the presence of PMeOx on the surface of the polyplexes, that acts as a protective colloid, hindering aggregation. The small size was also confirmed by AFM microscopy of the sample obtained at *R* = 15 (Figure 4c). This result is very important because, to the best of our knowledge, it is not common for systems of this architecture to achieve this small size. These results also underscore the importance of selecting appropriate materials that enable the formation of small drug delivery systems, suitable for environments with high viscosity and filtering barriers, such as pulmonary mucus.

Therefore, all these preliminary results indicate that the INU-VS-*g*-(PMeOx; bAPAE) copolymer has an excellent complexing ability of the genetic material, requiring low amount to complex siRNA, giving polyplexes with a mean size smaller than 30 nm and surface charge lower than 20 mV; all these properties could confer potential ability to the polyplexes, once inhaled, to penetrate both the mucus layer and the periciliary fluid, which has an estimated pore size of ∼40 nm.^48^

### 2.3 Stability of Polyplexes in Lung Fluids

To evaluate the suitability of INU-VS-*g*-(PMeOx; bAPAE)-based polyplexes for local lung administration by inhalation, we investigated their stability in the presence of mucus and lung surfactant components.

Since mucins, glycoproteins that constitute the main component of lung mucus, contain negatively charged sulfate and sialic acid groups,^49^ we assess whether a polyanionic exchange between mucins and siRNA could occur, potentially leading to the premature release of siRNA from the polyplexes before reaching the target site. In this context, two different mucin concentrations were tested, as the mucus composition in terms of the mucin concentration varies depending on the disease and its stage of progression. In particular, polyanionic exchange was evaluated by the electrophoretic assay in the presence of 1 and 5 mg mL^–1^ of mucin (Figure 5a,b, respectively), at *R* ratios higher than 5 (that is the minimum weight ratio that allows the formation of stable polyplexes).

**Figure 5:**
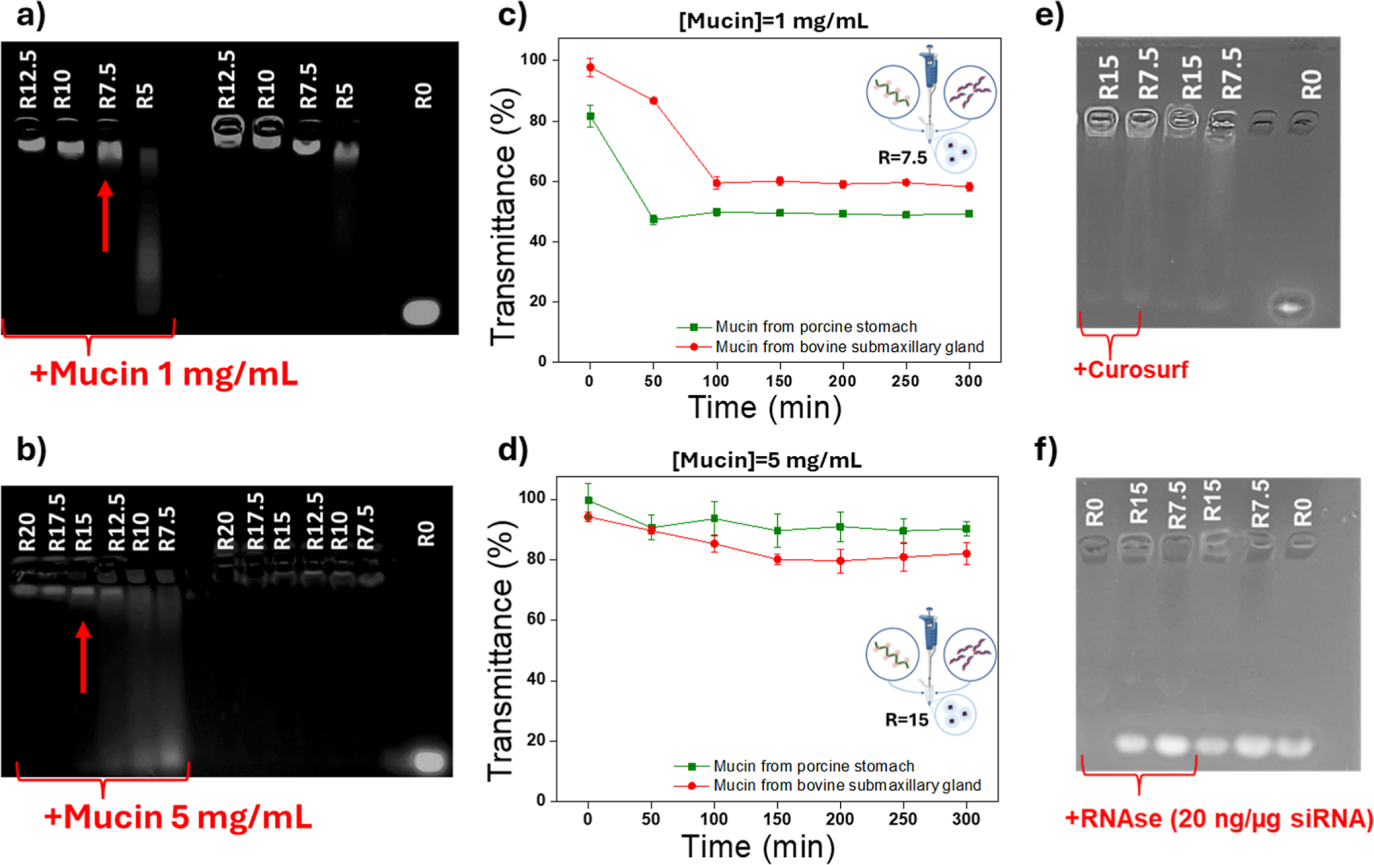
Evaluation of the electrophoretic mobility of siRNA in the polyplexes after 5 h of incubation with and without mucin at 1 mg mL^–1^ (a) and 5 mg mL^–1^ (b); transmittance at λ = 500 nm of mucin dispersions at 1 mg mL^–1^ in the presence of polyplexes *R* 7.5 (c) and at 5 mg mL^–1^ in the presence of polyplexes *R* 15 (d) (data reported as means SD, *n* = 3); and evaluation of the electrophoretic mobility of siRNA in the polyplexes after 5 h of incubation with and without Curosurf (e) and RNase (f).

As can be seen, when polyplexes were incubated with 1 mg mL^–1^ of mucins (Figure 5a), no polyanionic exchange occurred starting from a weight ratio equal to 7.5. This suggests high stability of these polyplexes in pathological conditions without significant mucin overproduction. Conversely, when the mucin content was increased 5-fold (Figure 5b), premature siRNA release from polyplexes was prevented starting from *R* = 15, corresponding to an amount of copolymer 3-fold higher with respect to a minimum complexation weight ratio. Therefore, these results suggest that polyplexes’ composition ratio can be properly modulated according to the pathological peculiarities, allowing their potential use also for the management of diseases characterized by excessive mucus secretion, such as cystic fibrosis (CF).^50^

Considering that mucins could not only give rise to polyanionic exchange but also may lead to the formation of aggregates with polyplexes, a turbidimetric analysis was conducted under experimental conditions where polyplexes were found to be stable (at *R* = 7.5 and *R* = 15, respectively, at 1 and 5 mg mL^–1^ of mucins), as a function of incubation time. Moreover, each turbidimetric assay was performed in the presence of two different mucins: the poorly water-soluble mucin from porcine stomach and the more water-soluble mucin from bovine submaxillary gland. Data, shown in Figure 5c,d, respectively, are expressed as the percentage of transmittance relative to the transmittance of a mucin dispersion, as a function of incubation time.

As observed, no substantial difference is detected between the two tests performed with mucin of different origins, indicating that the varying turbidity of the mucin dispersion does not affect the reliability of the assay. Regarding the polyplexes prepared at *R* = 7.5 and tested in a mucin dispersion at a concentration of 1 mg mL^–1^, the development of polyplex-mucin interactions is evident, as shown by the reduction in transmittance during the first 100 min, reaching values of approximately 50%. This trend is reversed for polyplexes prepared at *R* = 15 and tested in a mucin dispersion at a concentration of 5 mg mL^–1^, which, in contrast, do not appear to undergo significant polyplex-mucin interactions as no noticeable reduction in transmittance is recorded. Although this result may seem unexpected, as interactions generally increase with higher mucin concentrations, the polyplexes prepared at *R* = 15, containing a greater amount of polymer, exhibit a higher amount of PMeOx on their surface, which effectively shields interactions between the polyplexes and mucin chains, due to the formation of a thicker hydrophilic shell than that obtained with *R* = 7.5.

To confirm the muco-diffusive potential of obtained polyplexes due to the absence of interactions with mucins, a diffusion test was assessed using inserts for cell plates through a CF artificial mucus (CF-AM), showing rheological properties similar to those of pathological mucus.^21^ Polyplexes prepared at *R* = 15 were tested as these are stable in the presence of high mucin concentrations (Figure 5b), such as that present in CF-AM (i.e., 5 mg mL^–1^). To evaluate the effect given by the membrane of the inset, the simple diffusion was also studied under the same conditions but in the absence of CF-AM. The schematic representation of the test is reported in Figure 6a, while data, expressed as the amount of polyplexes found in the acceptor compartment (quantified by fluorescence), are shown in Figure 6b.

**Figure 6:**
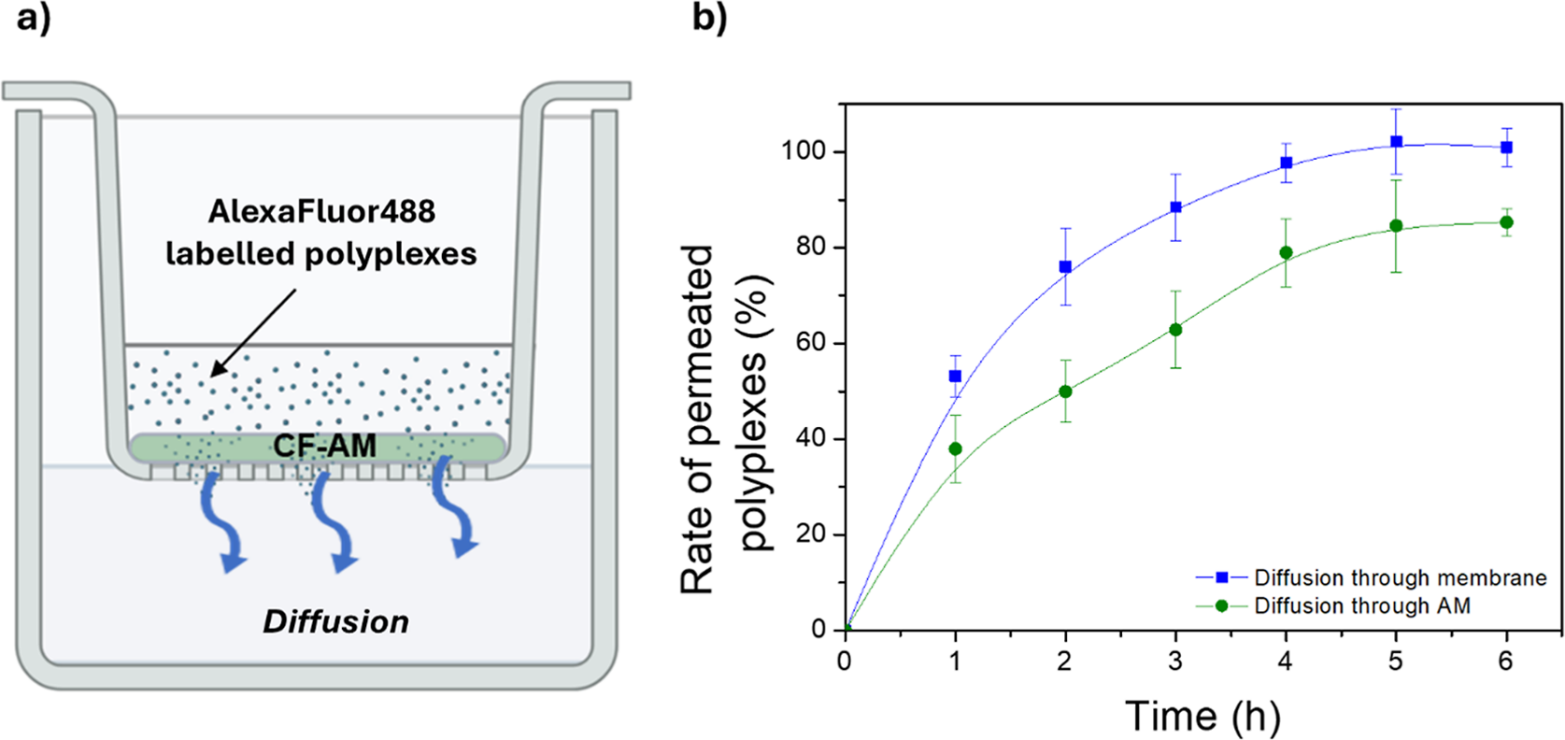
(a) Schematic drawing of the muco-diffusion experiment and (b) graphical rate of polyplexes (%) that reach the recipient compartment, compared with simple diffusion (data are reported as means SD, *n* = 3).

Results suggest that the presence of CF-AM does not hinder the muco-diffusion of polyplexes to the receiving compartment, as polyplexes reach the receiving compartment within 5 h in quantities corresponding to about 80% of the total.

Given that pulmonary barriers are not exclusively represented by the mucus lining the airways but also by the pulmonary surfactant, the stability of the polyplexes prepared at *R* = 7.5 and *R* = 15 was also evaluated in the presence of lung surfactant (Curosurf). As shown in Figure 5e, both polyplexes remained stable in the presence of lung surfactant, with no detectable release of siRNA after 5 h of incubation.

Similarly, considering that an efficient carrier should also protect nucleic acids from nuclease, the ability of polyplexes to protect siRNA from RNase-mediated degradation was assessed. As shown in Figure 5f, after the incubation with RNase A and the subsequent heparin-induced disassembly of polyplexes, the siRNA band remained still comparable to that of the respective control. In contrast, the band corresponding to noncarrier nucleic acid (*R*_0_) was completely absent, indicating total degradation of free siRNA.

Therefore, the siRNA complexation with the INU-VS-*g*-(PMeOx; bAPAE) allows to obtain polyplexes at low *R* values, stable in the presence of high mucin concentrations, able to diffuse toward the pathological mucus, and to protect siRNA from degradation, suggesting that the polyplexes can be administered by inhalation.

### 2.4 Biological Characterization of Polyplexes

Biological characterization on polyplexes was carried out on human bronchial epithelial cells (16-HBE) considering that, after inhalation, these are the first cells with which they come into contact, and which also represents a potential target for the management lung inflammation, being the site of production of specific pro-inflammatory cytokines.^13^ The cytocompatibility of the INU-VS-*g*-(PMeOx; bAPAE) copolymer, evaluated by MTS assay after 24 and 48 h of incubation in the presence of a copolymer concentration ranging from 0.005 and 0.5 mg mL^–1^, is reported in Figure 7a.

**Figure 7:**
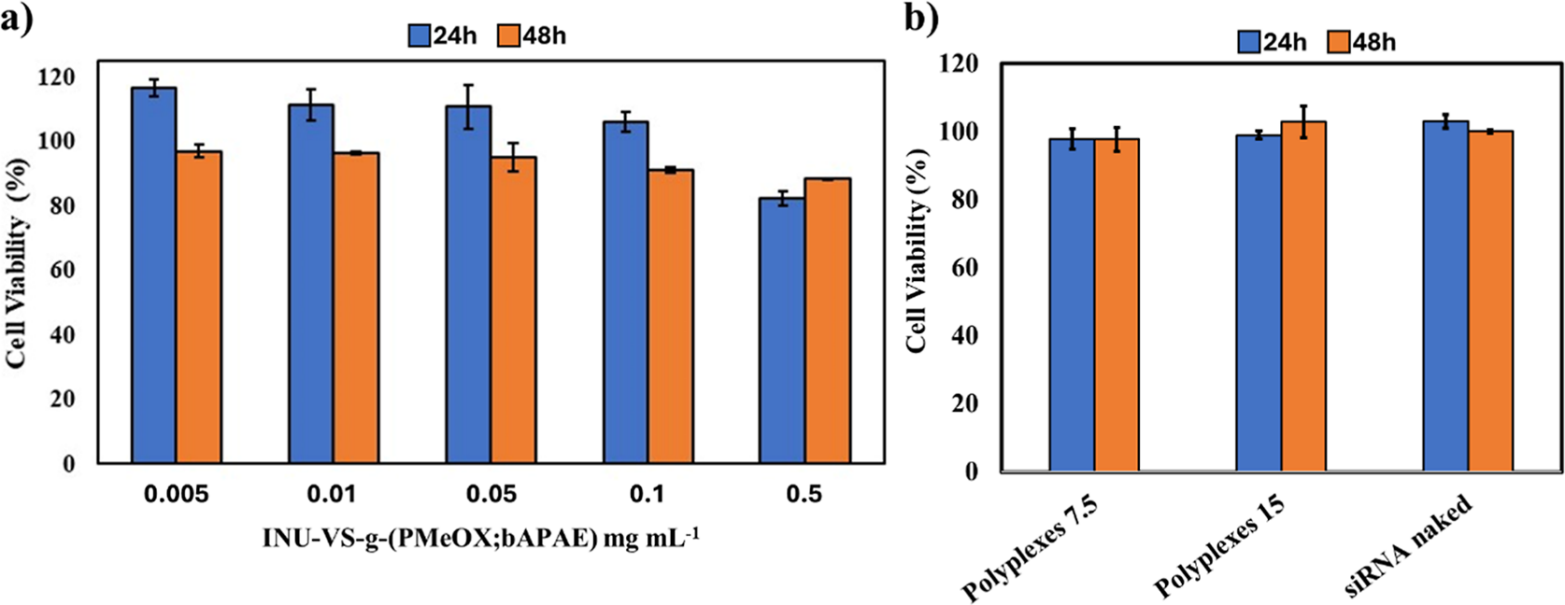
Cell viability on 16-HBE of (a) INU-VS-*g*-(PMeOx; bAPAE) graft copolymer at concentrations ranging between 0.005 and 0.5 mg mL^–1^ and (b) polyplexes at *R* = 7.5 and 15, after 24 and 48 h incubation.

Data indicate high cytocompatibility for INU-VS-*g*-(PMeOx; bAPAE), as cell viability remains at least 80% even after both 24 and 48 h of incubation in the presence of the highest copolymer concentration (0.5 mg mL^–1^).

In the same way, the cell viability assay was also carried out on cells incubated with proper amount of polyplexes to have a final concentration of siRNA equal to 100 nM (that is, the siRNA concentration typically used for the gene silencing assay), showing excellent cell viability values, both after 24 and 48 h of incubation (Figure 7b).

Since the target site of the siRNA is in the cytosol, the ability of these polyplexes to be endocytosed by the 16-HBE cells was assessed by fluorescence uptake studies by using polyplexes obtained from INU-VS-*g*-(PMeOx; bAPAE) labeled with Alexafluor488 and siRNA labeled with Alexafluor647.

As can be seen in Figure 8a, after 24 h of incubation, a fluorescence signal is detected following both copolymer and siRNA fluorescence; moreover, the localization of the two fluorescent probes is not entirely overlapping, suggesting a possible separation of siRNA from the polyplex in the intracellular environment. This separation could potentially make siRNA available to trigger the molecular mechanisms involved in RNA interference. On the contrary, no fluorescence signal was recorded in cells treated with naked siRNA.

**Figure 8:**
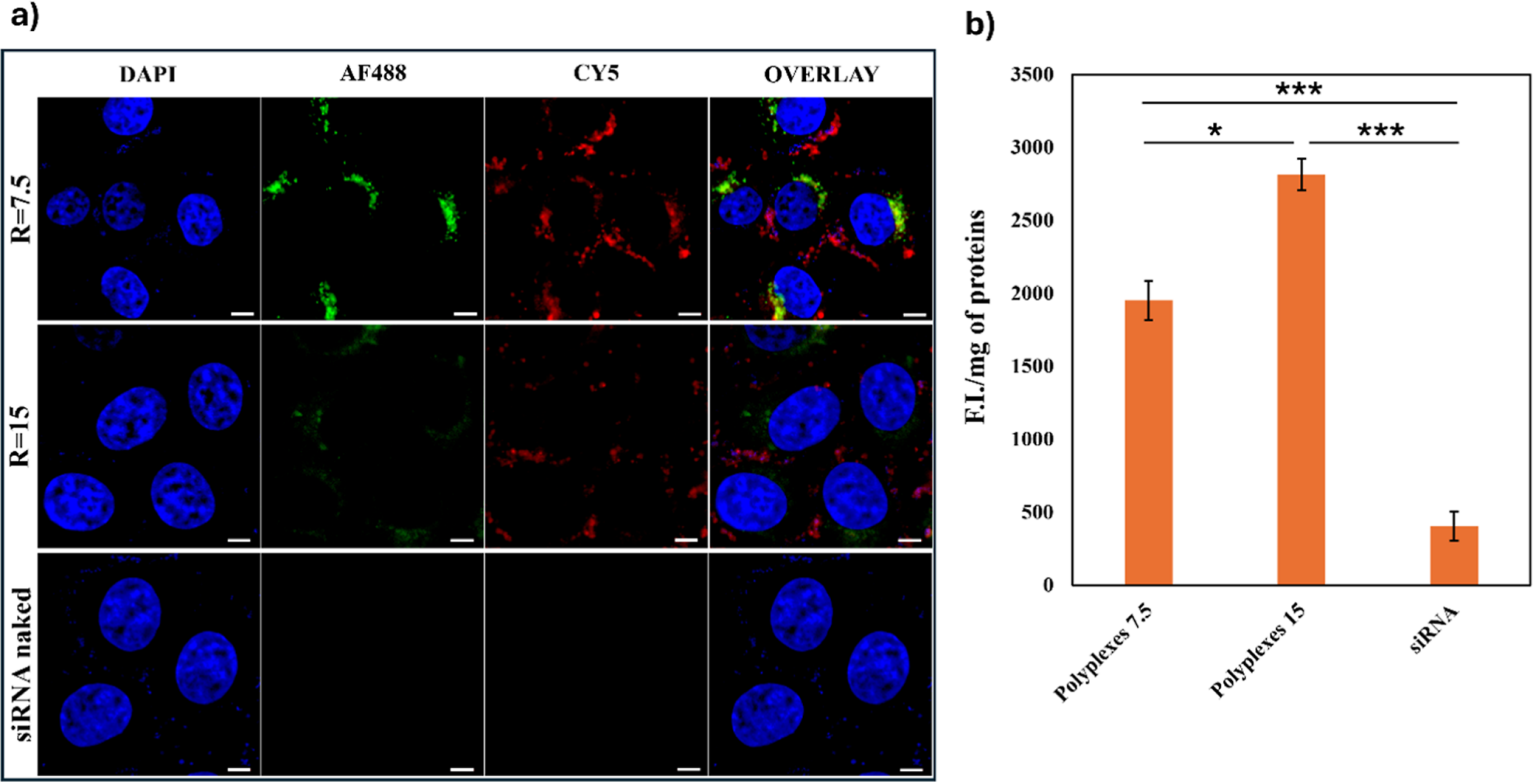
(a) CLSM images of 16-HBE and (b) cell uptake of INU-VS-*g*-(PMeOx; bAPAE)_Alexafluor488_/siRNA_CY5_ polyplexes at *R* 7.5 and 15 and naked siRNA, expressed as mean F.I./mg of protein, after 24 h of incubation. Bar represents 50 μm; data are expressed as means ± SD (*n* = 3) (P* < 0.05; P*** < 0.001).

The quantification of fluorescence of siRNA-Alexa647 contained in the cell lysate, reported in Figure 8b, confirms that siRNA involved in polyplexes was internalized more efficiently than free siRNA, the fluorescence being 5 and 7 times higher when cells were treated with polyplexes prepared at *R* = 7.5 and *R* = 15, respectively.

After demonstrating that these polyplexes exhibit good cytocompatibility and can be effectively internalized by bronchial epithelial cells, their gene silencing capacity was evaluated in vitro on MDA-MB-231 cells stably expressing the enhanced green fluorescent protein (eGFP) reporter gene as a model, using a siGFP as the siRNA model. Cells were treated with naked siRNA, polyplexes at *R* = 7.5 and 15, and a Lipofectamine–siRNA complex. Relative gene silencing (percentage) was calculated as (Fluorescence Intensity (F.I.) per mg of protein in treated cells/F.I. per mg of protein in positive control cells) × 100 and reported in Figure 9a.

**Figure 9:**
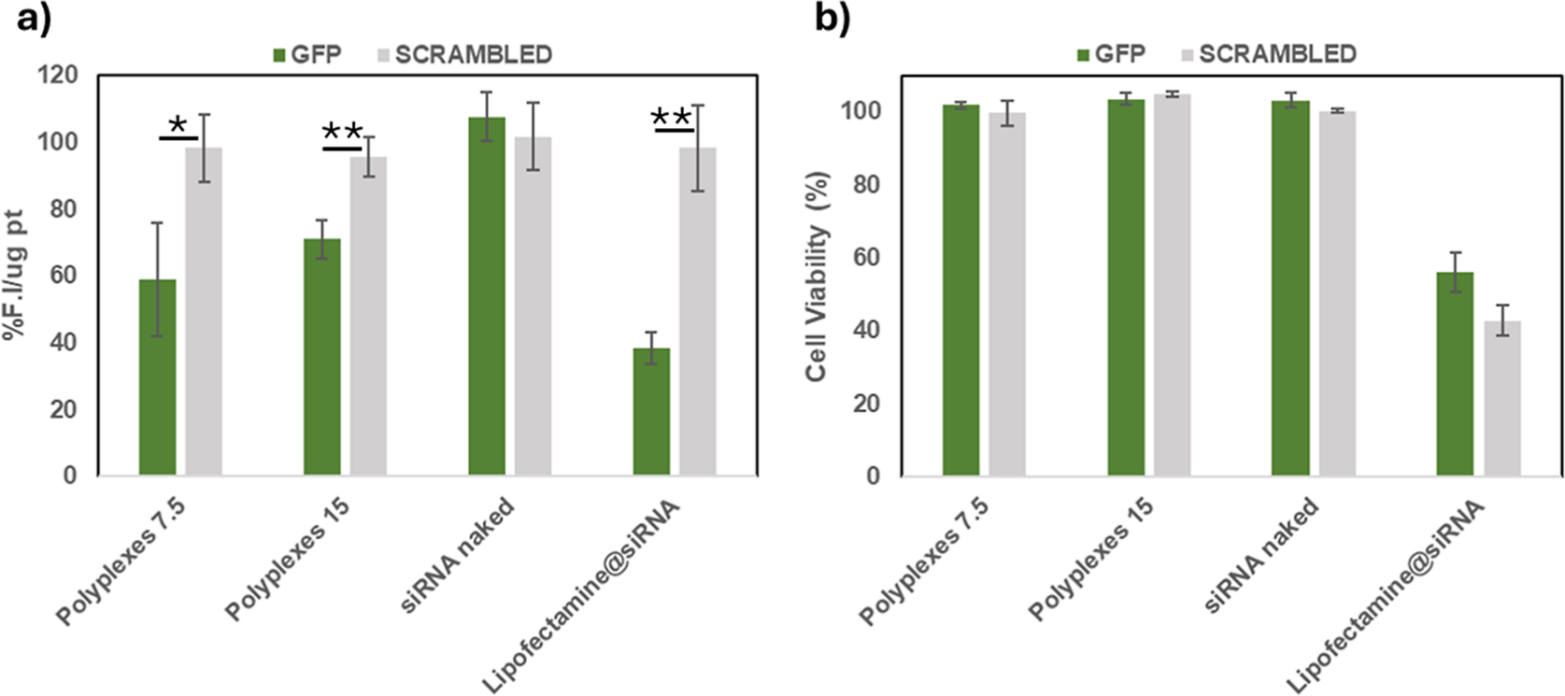
(a) Gene silencing, expressed as F.I. %/μg of protein and (b) cell viability on MDA-MB-231 cells after 48 h of incubation with polyplexes at *R* 7.5 and 15, naked siGFP or siRNA scrambled, and Lipofectamine@siRNA (100 nM siRNA/well). Data are expressed as means ± SD (*n* = 3). *p* > 0.05, p*** < 0.001.

The obtained results show that polyplexes achieved a significant silencing effect, with approximately 40% inhibition for polyplexes prepared at *R* = 7.5 and around 30% inhibition for those prepared at *R* = 15. This result can be explained by considering the varying ability of the siRNA to dissociate from the complex formed at *R* 15, before it can effectively interact with the target. In parallel, the same experiment was conducted using a scrambled siRNA sequence to prepare polyplexes, which are inactive, and no reduction in fluorescence was observed, confirming that the silencing effect is specifically attributed to the siGFP sequence. As expected, polyplexes were less efficient in silencing activity than lipofectamine. However, unlike lipofectamine, which reduces cell viability to approximately 50%, polyplexes do not cause a significant decrease in cell viability (Figure 9b).

These results successfully demonstrate the ability of the INU-VS-*g*-(PMeOx; bAPAE)-based carrier to release siRNA intracellularly and induce in vitro inhibition of eGFP gene expression, confirming its excellent potential as a vector for siRNA delivery to the lung.

### 2.5 In Vitro Pulmonary Drug Deposition

Finally, to evaluate the potential of the produced polyplex dispersion to penetrate the bronchial tree, we conducted an aerosolization performance test in vitro using an Andersen Cascade Impactor (ACI). Figure 10 illustrates the deposition profile of the drug across various ACI stages. The results indicated that approximately 40% of the inhaled dose was deposited between stage 3 and stage 7, corresponding to the bronchiolar and alveolar regions of the lungs. The formulation demonstrated efficient aerosolization, with a fine particle fraction (FPF) of 61.7 ± 0.28%, suggesting its suitability for deep lung deposition. The mass median aerodynamic diameter (MMAD) was measured at 3.91 ± 0.06 μm, while the geometric standard deviation (GSD) was 1.84 ± 0.04 μm, confirming that a significant portion of the dose falls within the respirable size range. Furthermore, DLS measurements performed on the sample recovered from the impactor did not reveal substantial changes in polyplex size, which remained approximately 27 nm, with a PDI of 0.328. These findings highlight the potential of the polyplex formulation for effective siRNA delivery to the lungs, supporting its applicability for inhalation-based therapeutic strategies.

**Figure 10:**
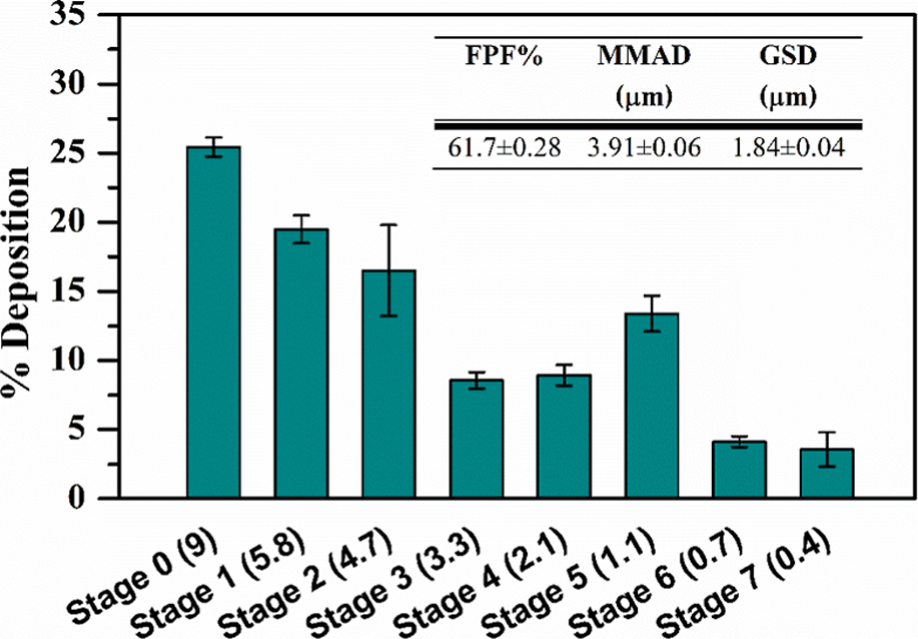
Deposition of polyplexes on the stages of the ACI.

## 3 Conclusions

In summary, we successfully synthesized and characterized a novel inulin (INU)-based copolymer, INU-VS-*g*-(PMeOx; bAPAE), designed for the pulmonary administration of siRNA via inhalation.

The very versatile synthetic procedure exploits the previous functionalization with divinyl sulfone (DVS) to obtain a hydrophilic and cationic copolymer in one pot, resulting in a very high-performance reaction in terms of grafting of amino groups and poly­(2-methyl-2-oxazoline) (PMeOx) chains. The developed copolymer exhibits a unique combination of polycationic and hydrophilic properties, with a well-defined balance between its amine-rich domains, responsible for siRNA complexation, and its hydrophilic PMeOx chains, which confer stability and prevent aggregation in physiological conditions.

The structural and functional characteristics of the copolymer, together with those of the siRNAs chosen as a model, allow self-aggregation to form small and poorly polydisperse structures, ideal for delivery in environments characterized by high viscosity and the presence of filtering structures.

Our findings demonstrate that this polymer not only efficiently complexes siRNA at low polymer/siRNA weight ratios but also forms stable polyplexes with sizes below 30 nm, which is an essential feature for effective penetration through the mucus barrier and periciliary fluid in the lungs. Moreover, the buffering capacity of INU-VS-*g*-(PMeOx; bAPAE) enhances endosomal escape through the proton sponge effect, which is a crucial step for intracellular siRNA delivery. The pH-dependent membrane destabilization further supports the hypothesis that this copolymer actively facilitates the cytosolic release of siRNA, improving its bioavailability and therapeutic potential.

Additionally, the polyplexes exhibit remarkable stability in the presence of lung mucus and surfactant, indicating their suitability for pulmonary administration. Importantly, the copolymer provides significant protection to siRNA against enzymatic degradation, ensuring prolonged bioactivity postadministration. Cellular uptake studies confirm efficient internalization of the polyplexes by bronchial epithelial cells, with a substantial increase in intracellular siRNA levels compared to free siRNA. This is further supported by promising in vitro gene silencing results on eGFP gene silencing using MDA-MB-231 GFP-modified cells as a model, demonstrating a significant inhibition of the target gene expression.

Taken together, these results highlight the potential of INU-VS-*g*-(PMeOx; bAPAE) as a highly promising siRNA carrier for pulmonary drug delivery applications for the management of lung diseases as it does not significantly interact with mucins and forms very small polyplexes capable of delivering siRNA via the inhalation route.

## 4 Experimental Section

### 4.1 Materials

Inulin (INU) obtained from Dahlia tubers (*M*_w_ = 5 kDa), divinyl sulfone ≥98.0%, dimethylformamide (DMF), methanol (MeOH), triethylamine (TEA), diethyl ether, dichloromethane, acetone, Dulbecco’s phosphate buffer saline (DPBS), NaOH, HCl, HEPES, 1,2-bis­(3-aminopropylamino)­ethane (bAPAE), benzonitrile, 2-methyl-2-oxazoline (MeOx), BOC-Piperazine, agarose, mucin obtained from pig stomach (Type II), mucin derived from Bovine Submaxillary Gland, deoxyribonucleic acid (DNA), diethylenetriaminepentaacetic acid (DTPA), RPMI 1640 Amino Acid Solution, egg yolk emulsion, NaCl, KCl, MISSIONsiRNA Fluorescent Universal Negative Control #1 Cyanine 5, and RNase A were obtained from Merck (Italy). Methyl trifluoromethanesulfonate (MeOTf) was obtained from Perlabo (Italy). Silencer Negative Control No. 1 siRNA (4404021) and Silencer GFP (eGFP) siRNA (AM4626), and AlexaFluor 488 NHS ester were purchased from Thermo Fisher (Italy).

The hydroquinone present in the commercially available divinyl sulfone ≥98.0% (used as stabilizing agent) was removed through neutral aluminum oxide column before use.

All materials used for biological characterization were purchased from Merck (Italy).

### 4.2 Cell Cultures

Human bronchial epithelial cells (16-HBE) were furnished by Istituto Zooprofilattico of Lombardia and Emilia Romagna. 16-HBE cells were cultured in a minimum essential medium [Dulbecco’s modified Eagle’s medium (DMEM)] (Euroclone, Milan, Italy) supplemented with fetal bovine serum (FBS, 10 vol %), l-glutamine (2 mM), amphotericin B (2.5 μg mL^–1^), streptomycin (100 μg mL^–1^), and penicillin (100 U mL^–1^) (Sigma-Aldrich, Milan, Italy). The breast cancer cells (MDA-MB-231) stably expressing green fluorescence protein (eGFP) were purchased from CliniSciences S.r.l. (Italy) and grown in the same culture medium used for 16-HBE supplemented with puromycin (0.6 μg mL^–1^).

All of the cell lines were left growing under standard conditions (relative humidity at 95%, 5% CO_2_, 37 °C).

### 4.3 Methyl-Poly­(2-methyl-2-oxazoline) Synthesis by Cationic Ring-Opening Polymerization (CROP)

Poly­(2-methyl-2-oxazoline)-Piperazine-Boc (PMeOx-Pip-Boc) to achieve a molecular weight equal to 5 kg/mol, as reported elsewhere.^51^ All reagents and solvents were treated with CaH_2_ and then distilled under vacuum and left under suitable storage conditions. Briefly, 360 mg of MeOTf (1 equiv) was introduced into a flask, previously dried and conditioned with Argon, and diluted with 42 mL of benzonitrile, to have a monomer’s concentration approximately to 3 M. After that, 10.7 g of MeOx (58 equiv) was added and the resulting mixture was left to stir at 120 °C for 3 h. After this time, the reaction was cooled and 1.23 g of 1-Boc-piperazine (3 equiv), dissolved in 3.5 mL of benzonitrile was added, and the resulting mixture was left to stir at 50 °C overnight. The polymer was finally isolated from the reaction mixture by precipitation in cold diethyl ether (0 °C); the suspension was thus centrifuged, and the solid precipitate was purified by washing twice with diethyl ether and dried under reduced pressure. The recovered product (obtained with a yield of about 97% by weight considering the initial quantity of monomer) was characterized by ^1^H NMR and SEC analyses.

^1^H NMR PMeOx-Pip-Boc (300 MHz, **CDCl**_**3**_, 25 °C, TMS): δ 1.44–1.46 (m, 9H, (C**H**_**3**_)_3_CO), 2.07–2.13 (m, 174H, [C**H**_**3**_CON]–), 2.94, 3.03–3.05 (m, 3H, C**H**_**3**_[N–C**H**_**2**_C**H**_**2**_]–), 3.45–3.47 (m, 232H [–C**H**_**2**_C**H**_**2**_N–]).

To remove BOC, 1 g of PMeOx-Pip-Boc was dispersed in 5 mL of HCl 4 M (200 mg mL^–1^) and was left to stir for 4 h at 25 °C, taking care to leave the reaction environment in communication with the outside. After this time, the temperature of the mixture was lowered on ice and 800 mg of solid NaOH (20 mmol) was dissolved in the solution; then, after solubilization of the NaOH, the solution was dried by a rotary evaporator. Then, 5 mL of MeOH was used to dissolve the solid product and the mixture was left to stir. After 15 min, the suspension was filtered on filter paper and the solution was added dropwise in cold diethyl ether (0 °C); the suspension was thus centrifuged and the residue dried under vacuum. The pure product (obtained with a yield of about 98% by weight considering the initial quantity of polymer) was characterized by ^1^H NMR and SEC analyses.

### 4.4 Synthesis of INU-Vinylsulfone (INU-VS)

1 g of INU (corresponding to 6.17 mmol of repeat unit) was dissolved in 20 mL of DMF. After complete dissolution, a 2.9 mL of purified divinyl sulfone (DVS) (corresponding to 30.85 mmol) was added and after few minutes, 4 mL of triethylamine (TEA) (corresponding to 30.85 mmol) was also added dropwise. The reaction mixture was heated to 60 °C and left to stir protected from the light for 24 h. After this time, the mixture was added dropwise in 200 mL of mixture (1:1), the resulting suspension was thus centrifuged, and the solid product was washed in diethyl ether/acetone five times. Then, the obtained solid was dissolved in ultrapure water (15 mL), filtered with a cellulose acetate filter (0.22 μm, Sartorius, Minisart Syringe Filter, Germany), and freeze-dried.

The final product, named INU-VS, was obtained with a yield of 92% w/w based on the starting INU, and then it was characterized by ^1^H NMR analysis and SEC analyses.

^1^H NMR INU-VS (400 MHz, **D**_**2**_ **O**, 25 °C, TMS): δ 3.62–4.0 (5H_INU_, m: –C**H**_**2**_ –OH; CH–C**H**_**2**_–OH; –CH_2_ –C**H**_**2**_ –O–), 4.12 (1H_INU_, t: C**H**–OH), 4.28 (1H_INU_, d: C**H**–OH), 6.41–6.52 (2H_VS_, m: C**H**_**2**_=) and 6.98 (1H_VS_, m: =C**H**–).

### 4.5 Synthesis of INU-VS-*g*-(PMeOx; bAPAE)

300 mg of INU-VS (corresponding to 1.434 mmol of repeat unit) was dissolved in 3 mL of ultrapure water. After complete dissolution, 360 mg of PMeOx previously solubilized in 1.5 mL of ultrapure water was added; the pH of the reaction was adjusted at 10 using 1 N NaOH and the mixture was protected from the light under stirring overnight. The following day the reaction mixture was added to a bAPAE solution (1 g in 75 mL of DMF) and the mixture was protected from the light under stirring overnight. After this time, part of the solvent was removed by a rotary evaporator, reducing the volume to about one-third and subsequently was precipitated in 250 mL of diethyl ether/DCM (2:1); the suspension was thus centrifuged, and the residue was washed with the acetone five times. Then, the obtained product was dried under vacuum, dissolved in 5 mL of ultrapure water and was further purified from byproducts by dialysis (MWCO 3.5 kDa) and then freeze-dried.

To obtain Alexa Fluor 488-labeled polymer, 1 mL of INU-VS-*g*-(PMeOx; bAPAE) 10 mg mL^–1^ in PBS pH 8.3 were mixed with 84 μL of Alexa Fluor 488 5-SDP Ester (2 mg mL^–1^ in DMSO) and left to stir for 1 h at room temperature. Then, the mixture was purified by dialysis (MWCO 3.5 kDa), and finally freeze-dried.^1^H NMR INU-VS-*g*-(PMeOx; bAPAE) (400 MHz, **D**_**2**_ **O**, 25 °C, TMS): δ 1.72 (m, 4 H_bAPAE_, NHCH_2_C**H**_**2**_CH_2_NHCH_2_CH_2_NHCH_2_C**H**_**2**_CH_2_NH), 2.11 (m, 174 H_PMeoX_, –[C**H**_**3**_CON]–), 2.65 (m, 12 H_bAPAE_, NHC**H**_**2**_CH_2_C**H**_**2**_NHC**H**_**2**_C**H**_**2**_NHC**H**_**2**_CH_2_C**H**_**2**_NH_2_), 3.11 (4 H_VS_, m: S–C**H**_**2**_–C**H**_**2**_–N) 3.56 (m, 232H_PMeoX_ [–C**H**_**2**_C**H**_**2**_N–]), 3.62–4.0 (5 H_INU_, m: –C**H**_**2**_ –OH; CH–C**H**_**2**_–OH; –CH_2_ –C**H**_**2**_ –O–), 4.12 (1 H_INU_, t: C**H**–OH), 4.28 (1 H_INU_, d: C**H**–OH).

### 4.6 Size Exclusion Chromatography (SEC)

SEC analysis was performed using a PolySep-GFC-P4000 column (PHENOMENEX) maintained at 30 °C, connected to an Agilent 1260 Infinity Multi-Detector GPC/SEC system, and a refractive index detector with buffer 0.15 M citrate/phosphate pH 5 as an eluent with a flow of 0.8 mL/min. The calibration curve was made by using poly­(ethylene oxide) standards.

### 4.7 Potentiometric Titration of INU-VS-*g*-(PMeOx; bAPAE)

30 mL of INU-VS-*g*-(PMeOx; bAPAE) (1 mg mL^–1^) was titrated under argon using 0.05 N HCl until pH 3. Then, the same mixture was titrated again with 0.05 N NaOH. The same conditions were used to titrate comparable amount of bAPAE. To stabilize ionic strength, 0.1 N degassed NaCl aqueous solution was added to the mixture to dissolve compounds. A Jenway differential electrometer was calibrated against a set of multiple standard buffers (2.50 ± 0.01 < pH < 10.00 ± 0.01) before to perform potentiometric titrations.

### 4.8 Membrane Destabilization Study

16-HBE cells were left growing at a cell density of 10.000 cells/well on an 8-well Nunc Lab-Tek Chambered Coverglass. The day after, the medium was withdrawn and to the cells 100 μL of INU-VS-*g*-(PMeOx; bAPAE) (5 μg mL^–1^) in DPBS (pH 7.4) or 20 mM MES (pH 5.5, 130 mM NaCl) was added. After 20 min at 37 °C, each well was washed with sterile DPBS, treated with 1 μM YO-PRO1 for 10 min at 37 °C, and fixed with 4 vol % formaldehyde in DPBS at room temperature. Cells were then observed with an inverted epifluorescence microscope (Axio Cam MRm, Zeiss), and the images were analyzed by using AxioVision software. In the same conditions, blank analysis was also performed using DPBS (pH 7.4) or 20 mM MES (pH 5.5, 130 mM NaCl).

### 4.9 Complexation Study

Complexation studies were evaluated by the gel retardation assay and by dynamic light scattering studies (DLS) analysis, as previously described.^30^ Polyplexes were prepared in 10 mM HEPES (pH 7.4) to achieve different polymer/siRNA weight ratios, by mixing equal volumes of the copolymer at different concentrations and siRNA 0.2 mg mL^–1^. The polymer/siRNA weight ratios annualized were 0, 1, 2, 3, 4, 5, 7.5, 10, and 15.

### 4.10 Atomic Force Microscopy

AFM micrographs were obtained on a FAST-SCAN microscope equipped with a closed-loop scanner (*X*, *Y*, and *Z* maximum scan regions: 35, 35, and 3 μm, respectively). Analysis was performed in soft tapping mode using a probe with an apical radius of 5 nm operating at 1400 kHz (*k*: 18 N/m).

### 4.11 Stability to Polyanionic Exchange in the Presence of Mucins

The stability of the polyplexes to polyanion exchange was determined after mixing polyplexes with different concentrations of mucin dispersion. The polyplexes were prepared as previously described, in order to obtain polymer/siRNA weight ratios (*R*) equal to 5, 7.5, 10, 12.5, 15, 17.5, and 20; after 30 min, the resulting polyplexes (15 μL) were mixed with same volume of mucin dispersion (2 mg mL^–1^ or 10 mg mL^–1^), in order to have a final mucin concentration of 1 mg mL^–1^ or 5 mg mL^–1^. After an incubation at 37 °C for 5 h, gel electrophoresis was then performed as described in the complexation study. As a control experiment, the same experimental conditions were repeated by replacing mucin dispersion with 10 mM nuclease-free HEPES buffer pH 7.4.

### 4.12 Evaluation of Polyplexes-Mucin Interactions

The evaluation of the possible interactions between polyplexes and mucins was carried out by the turbidimetric assay. 60 μL of polyplexes was prepared by mixing 30 μL of siRNA 0.1 mg mL^–1^ and copolymer solutions, obtaining a polymer/siRNA weight ratio (*R*) equal to 7.5 and 15.

Subsequently, 60 μL of mucin (Mucin from porcine stomach or Mucin from Bovine Submaxillary Gland) dispersion 2 mg mL^–1^ (in 10 mM HEPES buffer pH 7.4) was added to polyplexes prepared with *R* 7.5, while 60 μL of mucin dispersion 10 mg mL^–1^ (in 10 mM HEPES buffer pH 7.4) was added to polyplexes prepared with *R* 15.

After incubation at 37 °C, turbidity was measured every 50 min up to approximately 6 h. The absorbance at λ = 500 nm was recorded by the microplate reader.

Similarly, the absorbance at λ of 500 nm was recorded for polyplexes mixed with 60 μL of buffer to subtract the absorbance owing to scattering of the polyplexes and for mucin dispersion (1 mg mL^–1^ or 5 mg mL^–1^). Results were expressed as % of transmittance [(Abs500 mucins/Abs500 samples) × 100].

### 4.13 Muco-Diffusion of Polyplexes

To evaluate the ability of polyplexes to diffuse through the CF-AM layer, a donor/acceptor system was employed.^52^ CF artificial mucus (CF-AM) was prepared by mixing together 50 mg of DNA, 25 mg of Mucin from porcine stomach, Type II, 0.0295 mg of DTPA, 0.1 mL of RPMI 1640 Amino Acid Solution, 25 μL of egg yolk emulsion, 25 mg of NaCl, and 11 mg of KCl, in a final volume of 5 mL of DNase-free water.

MilliCell Cell Culture Insert 24-Well hanging Inserts (6.5 mm; pore size: 0.4 μm) were placed in 24-well plates containing 600 μL of 10 mM nuclease-free HEPES buffer (pH 7.4). A volume of 70 μL of CF-AM was placed on the membrane and then 30 μL of polyplexes (prepared with polymer/siRNA weight ratio (*R*) equal to 15, using siRNA 0.2 mg mL^–1^ and AlexaFluor 488 labeled copolymer) was deposited on the CF-AM layer. The system was incubated at 37 °C under continuous stirring (50 rpm). The fluorescence intensity at λ of 520 nm in the acceptor medium was measured hourly for up to 5 h using a microplate reader, with excitation at 480 nm.

For comparison, the experiment was repeated by replacing the CF-AM layer with a 10 mM nuclease-free HEPES buffer (pH 7.4). All experiments were performed in triplicate, and the results were expressed as the percentage (%) of total polyplexes permeated over time ± standard deviation (SD).

### 4.14 Stability of Polyplexes in the Presence of Pulmonary Surfactant

The stability of polyplexes in the presence of pulmonary surfactant was evaluated by gel electrophoresis, using condition described elsewhere.^53^ To 20 μL of polyplexes, prepared as previously described to obtain polymer/siRNA weight ratios (*R*) equal to 7.5 and 15, 5 μL of Curosurf was added. After incubation at 37 °C for 24 h, gel electrophoresis was then performed as described in the complexation study. As a control experiment, the same experimental conditions were repeated by replacing Curosurf with 10 mM nuclease-free HEPES buffer pH 7.4.

### 4.15 Stability Test in the Presence of RNase

The stability of the polyplexes in the presence of RNase was evaluated by incubating the polyplexes with RNase A at a RNase/siRNA weight ratio of 1:50. To 10 μL of polyplexes, prepared as previously described to obtain polymer/siRNA weight ratios (*R*) equal to 7.5 and 15 (corresponding both to 2 μg of siRNA), 2 μL of RNase A (20 μg mL^–1^) was added. The mixture was incubated at 37 °C for 1 h. Following this, 5 μL of 2% SDS was added, and the solution was incubated at 37 °C for an additional 10 min. Subsequently, 4 μL of heparin (1000 IU mL^–1^) was introduced, and a final incubation at 37 °C for 10 min was performed. The stability of the polyplexes was then analyzed by using gel electrophoresis, as described in the complexation study. For the control experiment, the same procedure was carried out, replacing RNase A with 10 mM nuclease-free HEPES buffer at pH 7.4.

### 4.16 Biological Characterization

#### 4.16.1 MTS Cell Viability Assay

Cell viability of 16-HBE cells was assessed by using an MTS assay with a commercially available kit (Promega). Cells were seeded at a density of 20,000 cells per well in 96-well plates. The day after, medium was replaced with 200 μL of the INU-VS-*g*-(PMeOx; bAPAE) copolymer in OPTI-MEM at concentrations between 0.5 and 0.005 mg mL^–1^. To ensure sterility, samples were filtered through a 220 nm membrane before incubation. After 24 and 48 h of incubation, wells were washed with sterile DPBS, and cells were incubated with 100 μL of fresh DMEM and 20 μL of MTS solution. After 2 h at 37 °C, absorbance at 490 nm was measured using a microplate reader (Multiskan Ex, Thermo Labsystems, Finland). Cell viability (%) was calculated as (Abs490 treated cells/Abs490 control cells) × 100, based on three independent experiments. Cells exposed to OPTI-MEM alone served as negative controls. In the same way, cell viability was evaluated after 24 and 48 h of incubation with 200 μL of OPTI-MEM containing INU-VS-*g*-(PMeOx; bAPAE)/siNC polyplexes, prepared at a weight ratio of 7.5 and 15 (100 nM siRNA per well).

#### 4.16.2 Cell Uptake Study

The cellular internalization ability of the polyplexes was evaluated by uptake studies on 16-HBE. Cells were plated with a density of 10,000 cells per well on an 8-well Nunc Lab-Tek Chambered Coverglass. After 24 h, the medium was replaced with 200 μL of OPTI-MEM containing INU-VS-*g*-(PMeOx; bAPAE) labeled with AlexaFluor 488/siRNA-Cy5 polyplexes, prepared at a weight ratio of 7.5 and 15, reaching a final siRNA concentration of 100 nM per well. After 24 h, cells were washed with DPBS and fixed with 4% formaldehyde. Cell nuclei were stained with 100 μL of DAPI (5 μg mL^–1^). After 5 min incubation, cells were washed five times with DPBS before being imaged using an Olympus FluoView FV10i Confocal Laser Scanning microscope.

For the quantitative determination of cell uptake, cells were seeded in a 24-well plate at a density of 100,000 cells per well and incubated with 300 μL samples, prepared as described above.

Following 24 h of incubation, cells were thoroughly washed with sterile DPBS and lysed in 100 μL of lysis buffer (2% SDS, 1% Triton X-100 in sterile DPBS). Fluorescence intensities of lysates were measured with a Shimadzu RF-5301PC spectrofluorophotometer (λ_ex_/λ_em_ 480 nm/520 nm for AlexaFluor 488 dye; λ_ex_/λ_em_ 649 nm/670 nm for Cy5 dye); the total protein content was evaluated via the BCA assay on 25 μL of lysates. The results were expressed as the fluorescence intensity normalized to the protein concentration. Naked siRNA-Cy5 and untreated cells (blank) were used as negative controls. Experiments were performed in triplicate. All reagents were sterilized by filtration through a 220 nm cellulose acetate filter before polyplex preparation.

#### 4.16.3 Gene Silencing Study

MDA-MB-231/eGFP cells were seeded in 48-well plates (30,000 cells in 0.3 mL medium per well) and transfected for 48 h with 300 μL of OPTI-MEM per well containing INU-VS-*g*-(PMeOx; bAPAE)/siGFP and INU-VS-*g*-(PMeOx; bAPAE)/siNC polyplexes, prepared at a weight ratio of 7.5 and 15 (100 nM siRNA per well), respectively. Lipofectamine_2000_ formulated with siGFP and siNC was used as positive controls, and naked siGFP and siNC were used as negative controls.

After this time, the transfected cells were washed with DPBS twice and lysed in 100 μL of lysis buffer (1% Triton X-100 in sterile water). Fluorescence intensity of lysates was measured with a microplate reader (Multiskan Ex, Thermo Labsystems, Finland) at λ_ex_/λ_em_ 480/520 nm while the total protein content was evaluated via the BCA assay on 25 μL of lysates. The results were expressed as fluorescence intensity normalized to the protein concentration. Experiments were performed in triplicate. In the same way, MDA-MB-231/eGFP cells were seeded in 96-well plates (15,000 cells in 0.15 mL medium per well) and incubated for 48 h with 150 μL of samples per well. After this time, the cells were washed with DPBS, and the cell viability was evaluated as described above.

#### 4.16.4 In Vitro Pulmonary Drug Deposition

The aerosolization performance of the aqueous dispersion of polyplexes was assessed in vitro, using an Andersen Cascade Impactor (ACI) (InPharmaTEC, Cogliate (MB), Italy). Before use, the ACI was refrigerated at 4 °C for 90 min to cool the equipment and minimize heat-transfer evaporative effects on the nebulized droplets, which could lead to droplet shrinkage and affect deposition behavior.^54^ The air-jet nebulizer with the mouthpiece was positioned toward the induction port using an adapter, while the ACI was connected to the vacuum source (Bavo X BIO, TCR TECORA, Italy). For the nebulization studies, 2.5 mL of polyplexes, containing 200 μL of polyplexes, prepared as previously described in order to obtain INU-VS-*g*-(PMeOx; bAPAE) labeled with AlexaFluor 488/siRNA weight ratios (*R*) equal to 15, was added to the sample chamber of the device. Nebulization was carried out for 7 min using the ACI in the 29 L/min configuration (Table 2 presents the aerodynamic cutoff diameters for the ACI stages). The pump was set to 4.5 L/min to ensure fraction deposition across the various ACI stages.

**Table 2:** Cutoff Aerodynamic Diameter for Stages of Andersen Cascade Impactor (ACI) at a Flow Rate of 29 L/min

ACI stages	cutoff diameter at 29 L/min(μm)
0	9
1	5.8
2	4.7
3	3.3
4	2.1
5	1.1
6	0.7
7	0.4

Subsequently, the polyplex dispersion deposited in each stage was recovered using 1 mL of water, and fluorescence intensities were measured by recording emission at 515 nm following excitation at 490 nm. Polyplexes’ dispersions at different concentrations were used as standards. Each experiment was performed in triplicate, and the resulting data were analyzed to determine the fine particle fraction (FPF %), defined as the percentage of the emitted dose with an aerodynamic diameter smaller than 5.0 μm. This was calculated by interpolating the cumulative diameter distribution curve. Additionally, the mass median aerodynamic diameter (MMAD) and geometric standard deviation (GSD) of the aerodynamic particle diameter were determined.

## Supplementary Material



## Notes

^1^H NMR spectrum (300 MHz, 298 K, CDCl_3_) of PMeOx-PipBoc with signal assignment and forward titration of INU-*g*-(PMeOx; bAPAE) and bAPAE (PDF)
