# Supplementary material for: Smart Inhalation Therapy: Boosting siRNA Efficacy with Inulin-Based Multifunctional Polymers
Source: ACS Appl Mater Interfaces. 2025 Oct 31;17(45):62647–61. doi: 10.1021/acsami.5c18977 (PMC12616608; doi:10.1021/acsami.5c18977)
Supplement: Supplementary file 1 [file am5c18977_si_001.pdf]

SUPPORTING INFORMATION

Smart Inhalation Therapy: Boosting siRNA Efficacy with Inulin-Based Multifunctional Polymers

Salvatore E. Drago, Marta Cabibbo, Cinzia Scialabba, Emanuela F. Craparo*, Gennara Cavallaro

Lab of Biocompatible Polymers, Department of Biological, Chemical and Pharmaceutical Sciences and Technologies (STEBICEF), University of Palermo, Via Archirafi 32, Palermo, 90123, Italy

*Corresponding author : emanuela.craparo@unipa.it

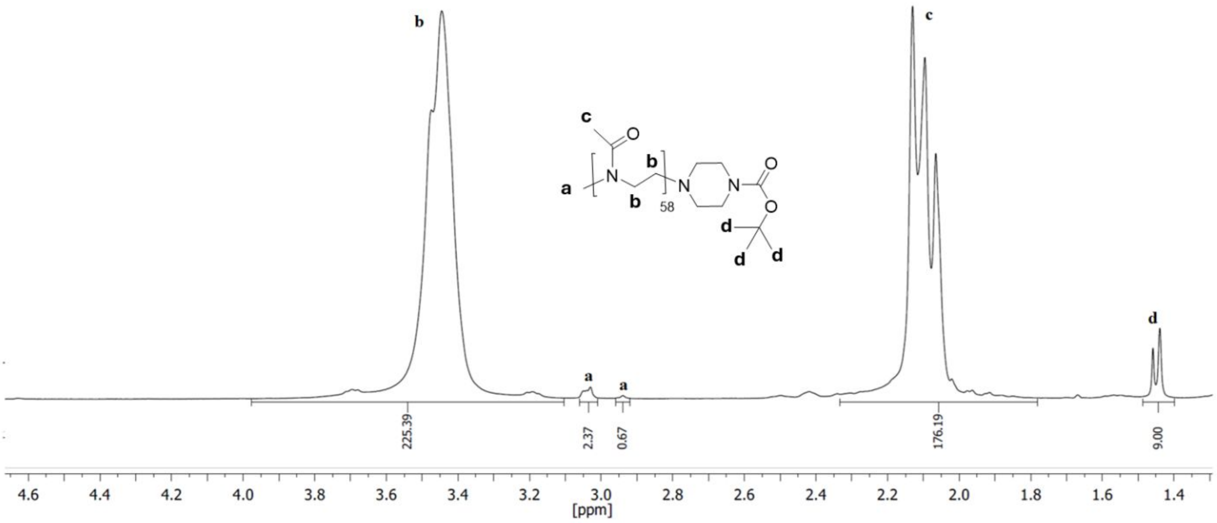

Figure S1. ^{1}H-NMR spectrum (300 MHz, 298K, $CDCl_3$) of PMeOx-PipBoc with signal assignment.

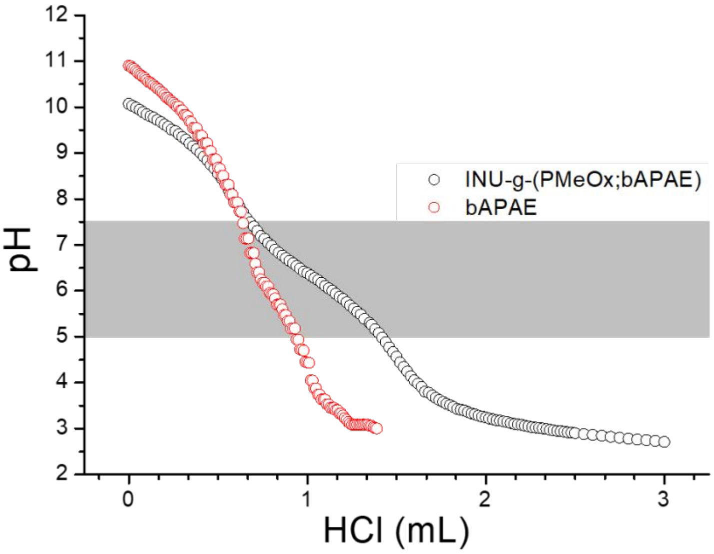

Figure S2. Forward titration (HCl volume versus pH) of INU-g-(PMeOx;bAPAE) (black circle) and bAPAE (red circle).
